# Eye-movement desensitisation and reprocessing therapy (EMDR) to prevent transition to psychosis in people with an at-risk mental state (ARMS): mixed method feasibility study

**DOI:** 10.1192/bjo.2024.57

**Published:** 2024-05-09

**Authors:** Daniela Strelchuk, Nicola Wiles, Katrina Turner, Catherine Derrick, David Martin, Jonathan Davies, Stan Zammit

**Affiliations:** Centre for Academic Mental Health, Population Health Sciences, Bristol Medical School, University of Bristol, UK; and National Institute for Health Research Bristol Biomedical Research Centre, University Hospitals Bristol and Weston NHS Foundation Trust and University of Bristol, UK; Centre for Academic Mental Health, Population Health Sciences, Bristol Medical School, University of Bristol, UK; National Institute for Health Research Bristol Biomedical Research Centre, University Hospitals Bristol and Weston NHS Foundation Trust and University of Bristol, UK; and Centre for Academic Primary Care, Population Health Sciences, Bristol Medical School, University of Bristol, UK; Centre for Academic Mental Health, Population Health Sciences, Bristol Medical School, University of Bristol, UK; Centre for Academic Mental Health, Population Health Sciences, Bristol Medical School, University of Bristol, UK; and Medical Education, Avon Wiltshire Partnership NHS Mental Health Trust, Bath NHS House Combe Park, Bath, UK; Centre for Academic Mental Health, Population Health Sciences, Bristol Medical School, University of Bristol, UK; National Institute for Health Research Bristol Biomedical Research Centre, University Hospitals Bristol and Weston NHS Foundation Trust and University of Bristol, UK; and MRC Centre for Neuropsychiatric Genetics and Genomics, Division of Psychological Medicine and Clinical Neuroscience, Cardiff University, UK

**Keywords:** EMDR, ARMS, psychosis, prevention, feasibility study

## Abstract

**Background:**

Trauma plays an important role in the development of psychosis, but no studies have investigated whether a trauma-focused therapy could prevent psychosis.

**Aims:**

This study aimed to establish whether it would be feasible to conduct a multicentre randomised controlled trial (RCT) to prevent psychosis in people with an at-risk mental state (ARMS), using eye-movement desensitisation and reprocessing therapy (EMDR).

**Method:**

This started as a mixed-method randomised study comparing EMDR to treatment as usual but, as a result of low participant recruitment, was changed to a single-arm feasibility study. The proposed primary outcome for an RCT was transition to psychosis at 12-month follow-up. Data on secondary outcomes were also collected. Qualitative interviews were conducted with patients and therapists.

**Results:**

Fourteen participants were recruited from the Early Intervention teams. Most people who expressed an interest in taking part attended an assessment to determine eligibility. All those eligible consented to take part. A total of 64% (7 of 11) of participants who were offered EMDR were followed up at 12 months. Of the 11 participants offered EMDR, one (11%, 95% CI: 0.2%, 48%) transitioned to psychosis. Nine patients and three therapists were interviewed. Participants who completed therapy (*n* = 4; mean 10.5 sessions) found EMDR helpful, but those who discontinued (*n* = 6; mean 5.2 sessions) said it had not benefitted them overall. Therapists said EMDR could be effective, although not for all patients.

**Conclusions:**

Future studies recruiting people with an ARMS to an RCT may need to extend recruitment beyond Early Intervention teams. Although some individuals found EMDR helpful, reasons for discontinuing need to be addressed in future studies.

Psychotic illnesses are some of the most disabling illnesses worldwide.^1^ The onset of psychosis is often preceded by a period of subthreshold psychotic symptoms, characterised as an at-risk mental state (ARMS) for psychosis.^2^ Of those identified as people with an ARMS, approximately 22% will make a transition to psychosis within a year and 36% within 3 years.^3^

Currently, there is limited robust evidence on preventing psychosis in people with an ARMS.^4,6^ National Institute for Health and Care Excellence (NICE) guidelines recommend cognitive–behavioural therapy (CBT) as a first line treatment.^7^ However, the meta-analysis underpinning these guidelines showed that CBT only had a moderate effect on transition to psychosis at 12-month follow-up (risk ratio 0.64, 95% CI 0.44, 0.93), and the benefit did not persist over the longer term (18-month follow-up: risk ratio 0.55, 95% CI 0.25, 1.19).^6^

## Trauma-focused therapy to prevent transition to psychosis

Exposure to trauma is one of the most well-established risk factors for psychosis. People with a history of trauma are two to four times more likely to experience psychosis.^8,10^ It has been hypothesised that pathophysiological mechanisms underlying post-traumatic stress disorder (PTSD) may also lead to the development of psychotic phenomena,^11,13^ with some empirical evidence to support this.^14,18^ Furthermore, a recent meta-analysis provided preliminary support for the role of trauma-focused therapies in reducing the positive symptoms of psychosis immediately after treatment (g = 0.31, 95% CI 0.06, 0.55).^19^ However, no studies have yet investigated whether psychosis could be prevented by helping people process their traumatic experiences.

Eye-movement desensitisation and reprocessing therapy (EMDR) is a highly effective trauma-focused therapy for PTSD,^20,21^ which does not require people to give a detailed description of their trauma, and therefore may be less distressing than trauma-focused CBT. The only trial of EMDR in people with psychosis and PTSD showed that EMDR improved PTSD and reduced paranoid delusions at 6-month follow-up.^22^

To investigate whether EMDR could prevent the onset to psychosis in people with an ARMS, a large multicentre RCT was needed. First, however, we needed to investigate whether such a trial would be feasible and acceptable to these individuals.

## Aims and objectives

The aim of this study was to establish the feasibility of conducting a large multicentre RCT that would examine the clinical effectiveness and cost-effectiveness of EMDR for the prevention of psychosis in people with an ARMS. The specific objectives were to:

- Estimate the rate of recruitment and retention for a large-scale RCT.
- Refine the eligibility criteria, screening and recruitment procedures.
- Explore patients’ and therapists’ views of EMDR as a treatment for ARMS, and their views on the study design and materials used.
- Optimise the EMDR protocol.
- Understand what treatment as usual (TAU) consists of for people with an ARMS. Findings related to this last objective are briefly presented in this paper, and further detailed elsewhere.^23^^,^^24^

## Method

### Study design

This was a single-arm feasibility study with a nested qualitative study, where all participants were offered EMDR. The study was originally designed as a randomised feasibility study comparing EMDR with TAU (see Supplementary material 1 available at https://doi.org/10.1192/bjo.2024.57 for detail on randomisation/blinding), but following low participant recruitment, the study design was changed to single-arm study. Detailed information about the study methods, including the change in design, is available in the published protocol.^25^ This feasibility study was conducted as part of a PhD thesis which explored the modifiable mechanisms underlying psychosis^26^.

The authors assert that all procedures contributing to this work comply with the ethical standards of the relevant national and institutional committees on human experimentation and with the Helsinki Declaration of 1975, as revised in 2008. All procedures involving human participants were approved by the South-West Cornwall and Plymouth Research Ethics Committee (Reference 18/SW/0037). Permissions to carry out the study were obtained from the NHS Trust involved. The trial registration was ISRCTN31976295.

### Inclusion/exclusion criteria

Eligible participants were individuals with an ARMS,^2^ aged 16 years or over, who had at least one positive symptom of psychosis scored ≥3 on the Comprehensive Assessment of At-Risk Mental State (CAARMS), a history of trauma occurring prior to the onset of the first positive symptom, and one or more PTSD symptoms during the last month (see Supplementary material 2 for more detail). Excluded were those with a history of treated or untreated psychotic illness or intellectual disability, currently taking antipsychotics or receiving psychological therapy, who had completed a trauma-focused psychological therapy in the past two years, with insufficient fluency in English or lacking mental capacity to provide valid informed consent.

### Recruitment

Recruitment took place in the Early Intervention teams of Avon and Wiltshire Mental Health Partnership NHS Trust (AWP) between 24 May 2018 and 31 May 2020. Recruitment ceased at the end of the predefined recruitment period, with participants followed up in line with the protocol. The recruitment procedure is described in full in the published protocol.^25^ In brief, those who were interested in the study and agreed to be contacted by the research team were invited to an appointment with the researcher to establish eligibility.

Potential participants were asked to complete the Life-Events Checklist for the Diagnostic and Statistical Manual of Mental Disorders, Fifth Edition (DSM-5 LEC),^27^ the Childhood Trauma Questionnaire (CTQ)^28^ and the PTSD Checklist for DSM-5 (PCL-5).^29^

Those eligible were asked to provide written consent for their participation. Sociodemographic information and medication use were collected using a self-report questionnaire. Additional baseline data were collected using quantitative measures covering: severity of psychotic symptoms (CAARMS,^2^ the Positive and Negative Syndrome Scale (PANSS) negative scale,^30^ the Psychotic Symptom Rating Scale (PSYRATS),^31^ Community Assessment of Psychic Experiences-42 (CAPE-42^32^), depression (Patient Health Questionniare-9 (PHQ-9^33^)) and anxiety (Generalized Anxiety Disorder-7 (GAD-7^34^)), functioning (Work and Social Adjustment Scale (WSAS^35^)), health status (EuroQol 5 Dimension 5 Level (EQ-5D-5L^36^)) and drug use (Drug Abuse Screening Test-10 (DAST-10^37^).

### The intervention: EMDR

Participants received up to 12 (90 min) sessions of weekly EMDR therapy. All were offered EMDR in person as originally planned, except for one individual who, in the context of COVID-19 restrictions, was offered EMDR online.

EMDR has been manualised and individualised to target psychotic symptoms^38,39^ by EMDR consultants from Lancashire and South Cumbria Care NHS Foundation Trust as previously described.^25^ Therapeutic sessions were delivered by four EMDR UK & Europe trained therapists recruited to the study and provided with training based on the treatment manual (Supplementary material 3). EMDR therapists received monthly in-person supervision with an EMDR UK consultant therapist. The average (mean) therapists’ age was 50.7 years (s.d. 4.7), two were male, and all had at least five years’ experience of delivering EMDR.

Therapeutic sessions were audio-recorded, and 10% (*n* = 6) were randomly sampled and evaluated by an accredited EMDR therapist independent of the study, to examine treatment fidelity (based on a five-point Likert scale: 1 = inadequate, 2 = weak, 3 = adequate, 4 = good, 5 = superior).^40^

### Data collection and outcome measurement

Outcomes were measured at 4, 8 and 12 months after the baseline assessment. The primary outcome was assessed at the 12-month follow-up, and the secondary outcomes at 4, 8 and 12 months.

Assessments were conducted by psychiatry trainees, psychology graduates and field workers with experience of conducting mental health assessments. All assessors received PANSS and CAARMS training (except for one experienced psychiatry trainee for whom it was not possible to provide in-Trust CAARMS training within an appropriate time frame because of unforeseen cancellations). All assessors had the option of discussing the assessments with the study chief investigator. Reliability checks were conducted as part of the CAARMS and PANSS training.

### Primary outcome measure

- Transition to psychosis: via the CAARMS at follow-up, or where participants did not attend their follow-up assessments via the International Classification of Diseases, Tenth Revision (ICD-10) diagnosis of psychotic disorder from clinical records.

### Secondary outcome measures

- Severity of psychotic symptoms (CAARMS, PANSS negative scale, PSYRATS, CAPE-42)
- Severity of PTSD symptoms (PCL-5)
- Severity of depression and anxiety (PHQ-9, GAD-7)
- Impaired functioning (WSAS)
- Health status (EQ-5D-5L)
- Drug use (DAST 10)
- Medication use
- Resource data use (see protocol for more detail).

More detail on instrument use/adaptation is provided (Supplementary material 4).

### Qualitative data

We conducted in-depth interviews with trial participants and therapists. Participants were interviewed after their 4-month follow-up or within a month of ending therapy. Therapists were interviewed after completing intervention delivery.

Topic guides were informed by our experience of interviewing patients and therapists about their views of receiving/offering psychological therapies as part of prior trials, and findings of other studies on trauma-focused interventions. Topic guides were discussed and agreed among the research team.

Topic guides were developed in parallel for patients and therapists to ensure key areas were discussed with both groups. This aided comparison of findings across the interviews, highlighting similarities and differences in patient and therapist views, and increasing the confidence with which study conclusions could be drawn. With interviewee consent, the interviews were audio-recorded and transcribed verbatim.

### Sample size

As this was a feasibility study, there was no formal sample size calculation. Following modification of our study design from a randomised to single-arm study, our target sample size was 20 participants.^25^

### Data analysis

#### Quantitative analyses

Quantitative data were analysed in Stata, version 16 for Windows (Stata Corp, College Station, Texas, USA; see https://www.stata.com/order/purchasing–faqs/). The statistical analysis plan was agreed in advance by the independent Trial Steering Committee.

The baseline characteristics of those recruited to the study were described (means [s.d.], or medians and inter-quartile ranges [IQR] if the distribution was skewed, for continuous variables, and numbers [%] for binary or categorical variables). As the primary interest was in recruitment and retention rates, we calculated the proportions of participants who (a) consented to do the baseline eligibility assessment, (b) completed the baseline eligibility assessment and agreed to take part in the study, and (c) completed the follow-up assessments at 4, 8 and 12 months – with 95% confidence intervals using the exact binomial method.

The proposed primary outcome (for a large-scale trial) was transition to psychosis at the 12-month follow-up. We reported the number and proportion of individuals who transitioned to psychosis at the 12-month follow-up. Given the change in design from a randomised to single-arm study, and hence the small number in the comparator group, it was not appropriate to provide a confidence interval around the effect size as originally specified.^25^ The completeness of data and summary statistics for each outcome measure and time point was compiled.

#### Qualitative analysis

Interview transcripts were analysed thematically.^41^ A subset of transcripts was independently coded by D.S. and K.T. After finalising coding frames, transcripts were imported into NVivo, version 12 for Windows (Lumivero LLC, Denver, USA; see https://lumivero.com/products/nvivo/) and coded electronically. Data were then analysed using an approach based on framework analysis,^42^ where data coded under specific codes were retrieved and summarised in tables. Summaries were read and reread, and comparisons made within and across the data to identify key themes and deviant cases and to understand participants’ views and experiences in relation to specific issues.

## Results

Supplementary material 5 and 6 provide detail on the recruitment challenges which led to a change in study design, and the impact of COVID-19 related restrictions on the study. For more detail on recruitment challenges, see also Strelchuck et al.^25^

### Recruitment

#### Recruitment to the original randomised feasibility study

All individuals who were identified as having an ARMS met the ARMS criteria according to the ‘Subthreshold attenuated psychotic symptoms’ criterion.

**Fig. 1:**
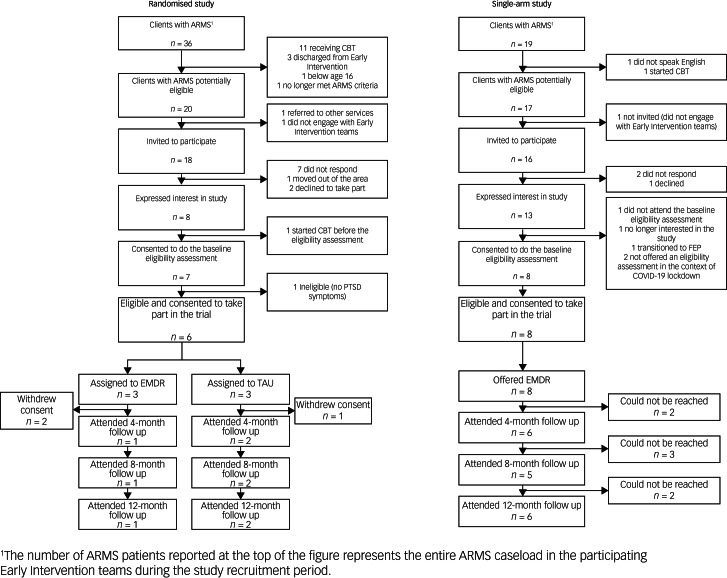
Consort flowchart to the randomised and single-arm study. ARMS, at-risk mental state; CBT, cognitive–behavioural therapy; EMDR, eye-movement desensitisation and reprocessing therapy; FEP, first episode psychosis; PTSD, post-traumatic stress disorder; TAU, treatment as usual.

Of 36 people identified as having an ARMS, 20 were potentially eligible, and 18 were invited to take part (Fig. 1). Of those invited, eight (44%) expressed an interest in participating. Seven individuals attended the eligibility assessment, of whom six (88%) were eligible (Table 1). All eligible participants gave written informed consent to take part in the study.

**Table 1:** Recruitment statistics

	Randomised study	Single-arm study
Total number of people with at-risk mental state (ARMS)	36	19
Number of people with ARMS potentially eligible	20	17
Total invited	18	16
Number who expressed interest (a)	8	13
**Baseline eligibility assessment**		
Number of participants who consented to do the baseline eligibility assessment (b)	7	8
Proportion who consented to do the baseline eligibility assessment:^a^ % (95% CI)	88 (47%, 99%)	62 (32%, 86%)
Number of eligible participants (c)	6	8
Number of eligible participants who consented to take part in the study (d)	6	8
Proportion who consented to take part in the study: % (95% CI)^b^	100 (54%, 100%^c^)	100 (63%, 100%^c^)

a. Proportion who consented to do the baseline eligibility assessment = b/a.

b. Proportion who consented to take part in the study = d/c.

c. One-sided, 97.5% CI.

#### Recruitment to the amended feasibility study

After changing to a single-arm study design following low participant recruitment, we identified a further 17 potentially eligible individuals. Of those, 16 were invited to take part, 13 expressed an interest and eight (62%) attended an eligibility assessment (Fig. 1) (Table 1). All participants were eligible and gave written informed consent for participation.

A total of 69% (*n* = 11) of participants were introduced to the single-arm study by their clinician, compared with only 22% (*n* = 4) in the randomised study. Those who were introduced to the study by their clinician were more likely to express an interest (14 of 15: 93% [95% CI 68%, 99%]), compared with those who were sent an invitation (7 of 19: 37% [95% CI 16%, 62%]).

#### Baseline characteristics

The baseline characteristics of all participants, grouped by study design and treatment offered, are shown in Table 2. Those who entered the randomised study (EMDR and TAU combined) (*n* = 6) and those who entered the single-arm study (*n* = 8) were similar in terms of sociodemographics and clinical characteristics. Not surprisingly for a small sample, there were some imbalances (e.g. with regard to clinician-reported hallucinations on PSYRATS). Given the small sample size, we combined data of all participants who were offered EMDR, and generated summary statistics.

**Table 2:** Baseline characteristics of study participants

	Randomised study			Single-arm study	All participants who were offered EMDR
	Eye-movement desensitisation and reprocessing therapy (EMDR)	Treatment as usual	Overall		
*N*	3	3	6	8	11
Age (years): mean (s.d.)	22.7 (2.7)	22.0 (3.1)	22.3 (1.8)	23.0 (1.6)	22.9 (1.3)
Male: *n* (%)	3 (100)	0	3 (50)	6 (75)	9 (82)
White: *n* (%)	3 (100)	3 (100)	6 (100)	6 (86)^1^	9 (90)^2^
Marital status: *n* (%)					
Single	3 (100)	3 (100)	6 (100)	7 (100)^1^	10 (100)^2^
Employment status: *n* (%)					
Student	1 (33)	1 (33)	2 (33)	−	1 (10)^2^
In paid employment (full or part-time)	−	1 (33)	1 (17)	2 (29)^1^	2 (20)^2^
Unemployed job seeker	−	1 (33)	1 (17)	2 (29)^1^	2 (20)^2^
Unemployed because of ill health	2 (67)	−	2 (33)	3 (42)^1^	5 (50)^2^
Ever worked: *n* (%)					
Full-time	2 (67)	1 (33)	3 (50)	3 (43)^1^	5 (50)^2^
Part-time	1 (33)	2 (67)	3 (50)	3 (43)^1^	4 (40)^2^
Never worked	−	−	−	1 (14)^1^	1 (10)^2^
Highest educational qualification: *n* (%)					
Higher National Certificate, Scottish Vocational Qualification or the Royal Society of Arts Higher diploma	−	−	−	1 (14)^1^	1 (10)^2^
Advanced (A) level, higher grade	1 (33)	2 (67)	3 (50)	4 (57)^1^	5 (50)^2^
General Certificate of Secondary Education, standard grade or other	2 (67)	1 (33)	3 (50)	2 (29)^1^	4 (40)^2^
Financial difficulty: *n* (%)					
Living comfortably/Doing all right	1 (33)	1 (33)	2 (33)	4 (57)^1^	5 (50)^2^
Just about getting by	1 (33)	2 (67)	3 (50)	2 (29)^1^	3 (30)^2^
Finding it difficult or very difficult to make ends meet	1 (33)	−	1 (17)	1 (14)^1^	2 (20)^2^
LEC score: *n* (%)					
Trauma happened to participant	3 (100)	3 (100)	6 (100)	7 (87.5)	10 (90.9)
Participant learned about it	−	−	−	1 (12.5)	1 (9.1)
CTQ score: mean (s.d.)	60.3 (13.5)	48.7 (6.2)	54.5 (7.1)	50.1 (5.7)	52.9 (5.4)
CAARMS score: mean (s.d.)					
CAARMS intensity	–^6^	–^6^	–^6^	11.5 (1.1)	11.5 (1.1)^3^
CAARMS frequency	–^6^	–^6^	–^6^	11.6 (1.5)	11.6 (1.5)^3^
CAARMS distress	–^6^	–^6^	–^6^	147.5 (28.7)	147.5 (28.7)^3^
SOFAS	–^6^	–^6^	–^6^	57 (5.5)	57 (5.5)^3^
PANSS – Positive scale: mean (s.d.)	18.0 (1.2)	14.0 (1)	16.0 (1.1)	–^4^	18.0 (1.2)^5^
PANSS – Negative scale: mean (s.d.)	17.3 (3.8)	17.0 (1)	17.2 (1.7)	14.8 (1.9)	15.5 (1.6)
PSYRATS score – Hallucinations: mean (s.d.)	22.3 (11.2)	20.0 (10.1)	21.2 (6.7)	16.6 (4.8)	18.2 (4.4)
PSYRATS score – Delusions: mean (s.d.)	8.0 (4.4)	7.3 (3.8)	7.7 (2.6)	9.8 (1.6)	9.3 (1.6)
Post-traumatic stress disorder checklist score: mean (s.d.)	62.3 (6.4)	52.7 (2.4)	57.5 (3.8)	58.5 (3.9)	59.5 (3.2)
CAPE-42 score: mean (s.d.)	37.3 (7.8)	38 (4.6)	37.7 (4.1)	39.9 (5.4)^1^	39.1 (4.2)^2^
PHQ-9 score: mean (s.d.)	21.3 (4.2)	16 (2.0)	18.7 (2.4)	19.4 (1.8)^1^	20 (1.7)^2^
GAD-7 score: mean (s.d.)	17.7 (0.9)	11.7 (3.5)	14.7 (2.1)	13.3 (2.3)^1^	14.6 (1.7)^2^
WSAS score: mean (s.d.)	26.3 (2.4)	16.3 (3.5)	21.3 (2.9)	23.6 (1.1)^1^	24.4 (1.1)^2^
EQ-5D-5L score: mean (s.d.)	11.7 (1.2)	10.3 (1.5)	11 (0.9)	10.7 (0.9)^1^	11.0 (0.7)^2^
DAST score					
Any drug: *n* (%)	2 (67)	2 (67)	4 (67)	1 (14)^1^	3 (30)^2^

LEC, LIFE Events Checklist; CTQ, Childhood Trauma Questionnaire; CAPE-42, Community Assessment Psychic Experiences; PHQ-9, Patient Health Questionnaire; GAD-7, Generalised Anxiety Disorder; WSAS, Work and Social Adjustment Scale; DAST, Drug Abuse Screening Test; CAARMS, The Comprehensive Assessment of At-Risk Mental States; SOFAS, The Social and Occupational Functioning Assessment Scale; PANSS, Positive and Negative Syndrome Scale; PSYRATS, Psychotic Symptom Rating Scale; EQ-5D-5L, EuroQol 5 Dimension 5 Level.

1 7 of 8 participants had complete data.

2 10 of 11 participants had complete data.

3 8 of 11 participants had complete data.

4 First study participants were assessed with the PANSS positive symptoms, as researchers in the study were not CAARMS-trained. Once researchers were offered CAARMS training, we switched from using the PANSS positive symptoms to CAARMS.

5 3 of 11 participants had data on this measure.

6 not applicable.

Eleven participants were offered EMDR as part of the randomised and single-arm study (Table 2). Mean age was 22.9 years (s.d. 1.3), and 82% were male. Approximately 90% were directly exposed to trauma, and the mean baseline PTSD score was 59.5 (s.d. 3.2). Mean PSYRATS hallucinations and delusions scores were 18.2 (s.d. = 4.4) and 9.3 (s.d. 1.6), respectively. Six participants (60%) reported that they were taking psychotropic medication (Supplementary material 7).

#### Completion of follow-up assessments

Of the 11 participants who were offered EMDR as part of the randomized and single-arm study, seven participants (64% [95% CI 30%, 89%]) were followed up at 4 months, six (55% [95% CI 23%, 83%]) at 8 months and seven (64% [95% CI 31%, 89%]) at 12 months (Table 3).

**Table 3:** Completion of follow-up assessments

	Randomised study						Single-arm study (*n* = 8)		All participants who were offered EMDR (*n* = 11)		
	Eye-movement desensitisation and reprocessing therapy (EMDR) (*n* = 3)		Treatment as usual (*n* = 3)		Overall (*n* = 6)						
	*n*	%	*n*	%	*n*	%	*n*	%	*n*	%	95% CI
4-month follow-up	1	33	2	67	3	50	6	75	7	64	30, 89
8-month follow-up	1	33	2	67	3	50	5	63	6	55	23, 83
12-month follow-up	1	33	2	67	3	50	6	75	7	64	31, 89

#### Therapy attendance

The mean number of sessions attended by all participants who received EMDR (*n* = 11) was 6.5 sessions (s.d. 1.3) (Table 4). Of the 11 participants, five (45%) received at least eight EMDR sessions which was regarded as a minimally adequate dose of therapy, and four (36%) completed therapy as intended (i.e. completed 12 sessions, or fewer if therapy goals were met earlier [average 10.5 sessions]). Six participants did not complete therapy (average 5.3 sessions; average 4.2 sessions after excluding one participant who stopped after 11 sessions due to an adverse event).

**Table 4:** Therapy attendance in all those who received eye-movement desensitisation and reprocessing therapy (EMDR) (in the randomised and non-randomised elements of the study)

	Randomised to EMDR (*n* = 3)	Offered EMDR as part of single-arm study (*n* = 8)	All participants who were offered EMDR (*n* = 11)
Number of sessions attended: mean (s.d.)	5 (1.7)	7.1 (1.7)	6.5 (1.3)
Received no sessions: *n* (%)	0	1 (13)	1 (9)
Received one to four sessions: *n* (%)	1 (33)	2 (25)	3 (27)
Received five to seven sessions: *n* (%)	1 (33)	1 (13)	2 (18)
Completed eight or more sessions: *n* (%, [95% CI])	1 (33)	4 (50)	5 (45 [2%, 80%])
**Outcome of therapy**^a^			
Completed therapy^b^: *n* (%, [95% CI])	1 (33)	3 (38)	4 (36 [11%, 70%])
Withdrew from therapy: *n* (%, [95% CI])	1 (33)	1 (13)	2 (18 [2%, 52%])
Discharged for non-cooperation: *n* (%, [95% CI])	0	1 (13)	1 (10 [0.2%, 41%])
Stopped therapy due to adverse events	1 (33)	1 (13)	2 (18 [2%, 52%])
Stopped therapy in the context of COVID-19 lockdown	0	1 (13)	1 (10 [0.2%, 41%])

a. This shows the outcome in people who were offered EMDR therapy (one participant never started).

b. This refers to participants who received 12 sessions or fewer if therapy goals were met earlier.

#### Treatment fidelity

The independent fidelity assessment found that 33% of therapy sessions rated were classified as ‘superior’, 33% as ‘good’, 17% as ‘adequate’ and 17% as ‘weak’.

#### Outcome data – proposed primary outcome

Eleven participants had data on the primary outcome measure, which was collected via the CAARMS for eight individuals and from clinical notes for the other three.

Of those who received EMDR and had available data at 12-month follow-up (*n* = 9), one participant (11%, 95% CI: 0.2%, 48%) had transitioned to psychosis. This participant had received only three therapy sessions as they paused therapy in the context of the COVID-19 restrictions, until in-person EMDR was again available. No individual from the TAU group (*n* = 2) transitioned to psychosis.

#### Outcome data – proposed secondary outcomes

Overall, 55% of participants (*n* = 6 out of 11) in the EMDR group and two out of three (66%) in the TAU group had complete data at 12-month follow-up on the scales that measured psychotic symptoms, PTSD or medication (Supplementary material 8). Data completeness was lower – approximately 45% – on the other secondary outcome measures in the EMDR group as, in the context of the COVID-19 restrictions, some questionnaires were posted to participants but not returned.

The mean scores on the secondary outcome measures and use of medication at the 4, 8 and 12-month follow-ups are shown in Supplementary materials 8 and 9.

Data collection pertaining to the use of healthcare services was split in two parts: a self-report questionnaire posted to participants, which was then followed up with a telephone call by a researcher. The completeness of the self-report questionnaire was approximately 55% in the EMDR group (Supplementary material 10) and 50% on the researcher-administered follow-up questions.

### Adverse events

All adverse events were assessed by the chief investigator. Serious adverse events (SAEs) were assessed for outcome, location, action taken and causality (i.e. not related/ unlikely/ possibly/ probably/ definitely related to the intervention/study procedures).

There was one serious and two non-SAEs in participants who were offered EMDR (Supplementary material 11), although frequent therapy contact made it more likely that adverse events were identified in this group. The SAE that had occurred was categorised as expected and, although unlikely to be related to the intervention, this possibility could not be excluded, and therefore, it was categorised as possibly related to the intervention.

### TAU in the participating teams

We recruited participants from the six Early Intervention teams in AWP. Of those, only three Early Intervention teams were funded to work with people with an ARMS and were able to offer them treatment. Treatment in these teams consisted of CBT for psychosis (CBTp), and in addition, one of the Early Intervention teams was also able to offer people family intervention and engage them in social groups. Early Intervention teams which were not funded to work with ARMS usually discharged patients back to their general practitioner (GP) or signposted them to non-statutory services. However, if individuals were suicidal or had a complex presentation, they were referred to Recovery Services.^24^

### Characteristics of participants interviewed

We interviewed nine individuals who took part in the study (two had been randomly assigned to usual care, and seven were offered EMDR as part of the randomised or single-arm trial). Of those offered EMDR, three completed therapy (referred to as ‘completers’) and four stopped early (‘non-completers’).

On average (mean), interviews lasted approximately 45 min. Five interviews were held by telephone, and four in person. Patients were aged 19 to 28 years (mean: 24.9 years [s.d. 3.8]), and two were female.

### Characteristics of therapists interviewed

We interviewed three of the four therapists who delivered EMDR. All interviews were conducted over the telephone. On average (mean), interviews lasted approximately 60 min. The average (mean) therapists’ age was 50.7 years (s.d. 4.7), and two were male.

### Interview findings

#### Participant interviews

Findings from participant interviews are organised under three main themes: (a) overview of EMDR sessions, (b) perceived impact of therapy and (c) views and experiences of taking part in the study.

##### Overview of EMDR sessions

Completers described how initially working on trauma was stressful, but as therapy progressed, they were able to process the trauma and look at it in a more detached way. One individual also described how processing trauma in EMDR was less distressing than in CBT. Those who discontinued therapy explained that they had difficulties connecting emotionally to the trauma, or on the contrary, were overwhelmed by the emotions associated with therapy.

It was just getting too much after a while and with everything else going on I couldn't relax, I couldn't control my anger … it was nothing to do with the session, it was just because … I'd have to then go home and deal with everything in my head … and I don't like where we live at the moment … it was quite hard to chill out afterwards. (EMDR non-completer 6)

##### Perceived impact of therapy

With regard to the impact of therapy, two of the three completers said EMDR had exceeded their expectations, and by the end of therapy their symptoms had almost gone.

I wasn't really expecting it [therapy] to go in and do as much as it did … to come to terms with what happened … and just be able to sort of move completely on from it. (EMDR completer 4)

However, the other completer said that although EMDR had improved their social functioning, it had not improved their mood.

The four non-completers reported that, overall, the few sessions they had received had not helped.

It [therapy] didn't have any effect on anything. (EMDR non-completer 3)

However, one non-completer also said that EMDR was better than any of the therapies they had tried in the past, as it had helped them feel more relaxed, but the timing at which they were offered EMDR was not ideal for them. Another non-completer said that, overall, they had enjoyed their therapy sessions as ‘sometimes it felt like a little bit of a weight had been lifted’ (EMDR non-completer 8), but other times it felt like they were revisiting memories which, in their view, did not need to be revisited.

##### Views and experiences of taking part in the study

When asked about their experience of taking part in the study, some participants said that participating had made them feel useful. Most said study documents were clear, albeit some said an audiovisual explanation would have been helpful.

#### Therapist interviews

Findings with therapist interviews are organised under two main themes: (a) trauma work and perceived effectiveness of EMDR in ARMS and (b) suggestions for modifying the EMDR protocol.

##### Trauma work and perceived effectiveness of EMDR in ARMS

Therapists said people were generally keen to start working on trauma, as they saw the connection between past experiences and current psychotic symptoms. However, some therapists explained that people with more complex trauma had difficulties making those connections and did not have the skills to regulate the distressing emotions associated with trauma processing.

Therapists were generally supportive of the effectiveness of EMDR in ARMS. One therapist said EMDR was highly effective and far exceeded their expectations, and another therapist explained that its effectiveness depended on the number of sessions received, how clearly defined the trauma was and the availability of positive resources to draw on.

It [EMDR]'s been an amazing experience for them [patients] … and it's exceeded all their expectations … So my experience is that it's been highly effective. (Therapist 3)

I think it can be really effective. I think it's quite variable really … if there were more sessions available to use EMDR I think it could have been much more effective … if there's … sort of clear trauma there then I think it can be really useful. (Therapist 2)

Another therapist said that they had limited experience of using EMDR for an ARMS, but in their view, the effectiveness of EMDR was not diagnosis-dependent, but it depended on individuals’ readiness for therapy.

I think my experience of it is too limited to say [how effective EMDR is] … that's not diagnosis dependent … It's all about preparation and support and understanding and a willingness, and the therapy relationship to enable that to kind of get the best outcome you can. (Therapist 1)

Two therapists viewed EMDR as less distressing than trauma-focused CBT, as it did not ask people to describe trauma in detail and did not involve any homework.

##### Suggestions for modifying the EMDR protocol

When asked about suggestions for modifying the EMDR protocol, therapists mentioned (a) increasing the number of available sessions, (b) ensuring therapists being trained in using techniques such as the Flash technique and taxing working memory tasks, to decrease individuals’ distress and facilitate trauma processing, and (c) offering individuals with attachment difficulties additional preparatory work before engaging them in EMDR.

Two therapists said EMDR may not have been a very good fit for those people who had less clearly defined trauma or had attachment difficulties, and raised the question of whether offering people EMDR based only on them belonging to a certain patient group (i.e. ARMS) would be a helpful approach.

## Discussion

### Summary

The overall aim of this study was to establish the feasibility of conducting a large multicentre RCT for the prevention of psychosis in people with an ARMS. We summarise below the findings corresponding to each objective, and present recommendations for future trials on ARMS.

The decision of whether to proceed to a future large multicentre RCT was judged based on this study's findings regarding the feasibility of recruiting and retaining participants, and the acceptability of EMDR to patients and therapists.

#### Objective 1: estimate the rate of recruitment and retention for a large-scale RCT

Most people who expressed an interest in taking part in the study agreed to attend a baseline assessment to determine eligibility, and all those eligible consented to take part. This indicates that EMDR could be an acceptable treatment for people with an ARMS. However, the low number of people identified as having an ARMS in the Early Intervention teams in AWP made it difficult to reach our recruitment target. With few exceptions (e.g. Early Intervention teams in North West England), the number of people with an ARMS in Early Intervention teams was generally low,^43^ which, in our view, suggests that (with the exception of those Trusts which have a well-established pathway into care for people with an ARMS^44^) recruiting people with an ARMS to a large multicentre RCT exclusively via the Early Intervention teams may be difficult in many Trusts in the UK.

Although most (9 out of 14) study participants were followed up at 12 months, there was considerable imprecision around the estimates of retention rates, given the small numbers.

The primary outcome was collected for 11 individuals. Given the difficulties with following up participants, it was important to have the option of collecting data from clinical notes. Approximately 50% of participants had complete data on the proposed secondary outcome measures, with those returned by post being more incomplete (e.g. PHQ-9, GAD-7, WSAS).

#### Objective 2: refine the eligibility criteria, screening and recruitment procedures

Our exclusion criteria of not offering people EMDR concurrently with other psychological interventions (e.g. CBTp) negatively impacted participant recruitment at the start of the study (i.e. the randomised stage). This was because at the start, we were only able to recruit from Early Intervention teams that were funded to work with ARMS, because of difficulties with securing excess treatment costs. As people from these teams were offered CBTp as part of TAU, most had already started CBTp by the time we opened recruitment and were therefore not eligible for our study. However, as the study progressed, Early Intervention teams did not start providing CBTp until after we had conducted eligibility assessments for new people with an ARMS so that eligible individuals could be offered EMDR, as part of the study, or CBTp as standard treatment. Recruitment was improved from 6 months in once we obtained funding to pay therapists working in a private capacity, enabling us to recruit from those Early Intervention teams that were not funded to work with ARMS. This explains the discrepancy in the reason for exclusion in the randomised versus single-arm part of the study. However, given the small number of people with an ARMS in the participating Early Intervention teams, we were unable to meet our recruitment target, and therefore recommend that future ARMS trials extend recruitment to primary care services.

We think that future ARMS trials should investigate the effectiveness of EMDR separately from other psychological interventions, as by offering clients EMDR and CBTp in parallel it would be difficult to disentangle the impact of the two interventions and would require a substantially larger sample size if evaluating EMDR as an adjunct to CBTp, compared with CBTp alone. Furthermore, offering people two psychological interventions such as EMDR and CBTp concomitantly would not usually happen in clinical practice, and therefore the findings of such a trial would be difficult to generalise to current clinical practice.

The impact of this exclusion criterion on recruiting participants from Early Intervention teams where CBTp is offered as part of TAU could be minimised by ensuring a close liaison between researchers and clinical teams, so that people have the option to participate in a trial in a timely fashion. Clinical teams could still offer other psychological therapies (e.g. CBTp) after completion of EMDR, if required.

#### Objective 3: explore patients’ and therapists’ views of EMDR as a treatment for ARMS, and their views on the study design and materials used

Qualitative interviews showed that all participants who completed therapy found EMDR helpful. Those who discontinued said EMDR had not benefitted them, although one non-completer also said that EMDR was better than other talking therapies they had received in the past. The low average number of sessions for those not completing therapy suggests that this may be because of factors other than lack of an effect of EMDR (e.g. timing of treatment, inability to connect emotionally to memories or feeling overwhelmed by emotions, all of which were reasons given for stopping therapy during interviews). Refinement in the protocol, training or supervision may be required to ensure participants are in stable environments and are well-enough resourced for them to start processing pathogenic memories in future trials. Therapists were generally positive about the effectiveness of EMDR in ARMS, although not for all clients.

#### Objective 4: optimise the EMDR protocol

Some therapists reported that (a) increasing the number of available sessions, (b) using the Flash technique and taxing working memory tasks to decrease patient distress and facilitate trauma processing and (c) offering additional preparatory work to people with attachment difficulties before the start of EMDR would be helpful in future trials of EMDR. In addition, it was also mentioned that increasing the number of therapy sessions would make EMDR more effective.

#### Objective 5: understand what TAU consists of for people with ARMS

Only half of the Early Intervention teams we had recruited from were funded to offer treatment to people with ARMS. TAU in these teams usually consisted of CBTp, although one of the teams was also able to offer family interventions and to engage the individuals in social groups. TAU in those Early Intervention teams which were not funded to work with ARMS usually consisted of discharge back to the GP, and sometimes (e.g. when a person's risk to self or others was high), referral to Recovery Services.

For a summary of the challenges we faced in our study and recommendations for future ARMS trials, see Supplementary material 12.

### Strengths and limitations

The biggest limitation of the study was the low number of people recruited. This meant that it was not possible to make meaningful comparisons between the EMDR and TAU group in terms of likely retention rates for a larger multicentre trial. As people with ARMS should be referred to the Early Intervention teams for specialist assessment and treatment,^7^ the Early Intervention teams were thought to be the right place for recruiting these individuals, and our recruitment processes aligned with this. Extending recruitment to primary care services (including Primary Care Liaison Services) would have exceeded the limited resources of this feasibility study.

The original plan was that all follow-up assessments would be conducted in-person as they were more likely to achieve higher follow-up rates. However, in the context of the COVID-19 restrictions, the follow-up assessments were conducted over the telephone. The latter were more challenging owing to technical issues (around connections/signal), and/or participants finding it difficult to answer questions over the telephone, which may have contributed to the substantial amount of missing data. That said, there was no clear difference between the 4-, 8- and 12-month follow-up assessments. Including the option of videocall assessments or filling out questionnaires online may improve completion rates in future studies.

Informal discussions with the independent assessor indicated that the scale used for assessing fidelity was quite laborious, suggesting that future studies may need to use a simplified version. In addition, there were difficulties with rating individual sessions as some items were not completed in that session and could therefore not be scored, even though it is likely they were covered in a previous session.

Given the limited number of study participants, we were only able to interview a small number of patients and therapists. Although this meant we were not able to reach data saturation, the interviews provided some important insights into patients’ and therapists’ views of EMDR. Participants in this study were recruited from the Early Intervention teams in AWP. Given the high thresholds for accessing secondary care services, it is possible that people who took part in this feasibility study had more severe symptomatology compared to the general ARMS population. In addition, the high variability in the level of support that the Early Intervention teams offered to their patients may have further contributed to these people's response to treatment or engagement with therapy.

Therapists’ discrepant views on EMDR may reflect the heterogeneity of this patient group, and therapists’ different levels of experience and expertise in working with people with ARMS. In addition, therapists had offered EMDR to a small number of individuals, and therefore had limited experience to draw upon. It is possible that other therapists with more experience of working with this patient group, or who had provided EMDR therapy to a larger number of people with ARMS, would hold different views from the ones presented here. It is therefore recommended that future studies further explore the views of people with ARMS and therapists about EMDR therapy.

The interviews were conducted by D.S., a PhD student, who prior to conducting the interviews had been involved in the baseline and follow-up assessments of participants and liaison work with therapists. Although this might have put participants at ease when describing their experiences of EMDR, it is also possible that they described their experiences in more positive terms than they would have otherwise done. In terms of the therapists, they too might have described their experiences in more positive terms, as they knew the interviewer was responsible for the study. That said, all interviewees were asked to be open about their opinions and were aware this was a feasibility study, and therefore the data highlighted both the benefits and challenges of offering EMDR to individuals with ARMS.

### Comparison with literature

To date, there are only two completed UK trials for the prevention of psychosis in ARMS.^45,46^ Both examined the effectiveness of CBT and randomised approximately 50% of those assessed for eligibility. In our study, the majority of those who were eligible agreed to take part. Hence, the main difficulty with recruitment in our study was not related to the acceptability of the study or intervention, but to the low number of people with ARMS in contact with Early Intervention teams.

Another feasibility study of individual and family CBT for ARMS mainly recruited from Early Intervention teams in North West England and received 173 referrals, of which 108 were assessed, and 70 randomised.^44^ Comparisons of the absolute number of people recruited could be misleading as this depends on the number of Early Intervention teams recruited from and the size of the catchment area. Therefore, if we compare the percentage of people recruited in relation to those referred, then recruitment to our study (14 recruited/37 referrals = 38%) is quite similar to Law et al.^44^ (70 recruited/173 referrals = 40%).

The retention rates at 12-month follow-up (~60%) in this study are comparable with those reported by Morrison et al (~65%).^46^ However, given the small sample size, there was considerable uncertainty around our estimate.

The transition rate in our study (although imprecise) is comparable to findings of other trials. For example, Van der Gaag et al^47^ found transition rates of 11% in the CBT group and 22% in the TAU group at the 18-month follow-up, and Morrison et al^46^ found 6.9% in the CBT group and 9% in the TAU group at the 24-month follow-up. However, given the relative arbitrariness of the threshold for transitioning to psychosis, future studies may want to use – as the primary outcome – the severity of psychotic symptoms rather than a binary measure of transitioning to psychosis.

Clinicians have generally been reluctant to offer trauma-focused therapies to people with psychosis.^48^ One reason for this is that clinicians fear that addressing trauma may lead to an increase in the severity of positive symptoms or suicidal behaviour.^49^ Our findings expand the research available so far on the application of trauma-focused therapies in people with psychotic symptoms^50,52^ and show that EMDR can be acceptable and helpful for people with ARMS.

All those who completed therapy as part of our study said EMDR was helpful, with some of them reporting that EMDR had exceeded their expectations and helped them come to terms with their past. Our findings are consistent with those of a recent systematic review which showed that clients who received EMDR described their therapeutic experience in a transformational manner.^53^ However, the fact that some participants in our study discontinued therapy suggests that either the timing at which they were offered therapy was not ideal for them (e.g. some did not have a secure place to live), or that they might have needed more preparatory work before moving to the trauma-processing stage. Consistent with our results, a qualitative study exploring the experiences of trauma-focused imaginal exposure for voice hearing found that people's therapy experience can be negatively affected by outside circumstances,^52^ and highlighted the need for a supportive environment outside of therapy. Others have also highlighted the importance of offering trauma-focused interventions in a multidisciplinary setting.^51^ Regarding the value of preparatory work prior to trauma processing, a case series study of imaginal exposure for trauma-related hallucinations showed that some people would have benefitted from stabilisation work prior to engaging in exposure.^54^ However, the need for additional preparatory work is disputed, with some researchers arguing that emotion regulation difficulties do not affect treatment outcomes in people with severe PTSD.^55,56^

Some participants reported the recollection of past traumatic memories during EMDR as overwhelming, or mentioned that they had difficulties relaxing outside the session. However, there are techniques within the EMDR framework which can prevent their distress levels from becoming too high for them to engage effectively with trauma processing (e.g. the Flash technique^57^ and taxing memory tasks^58^), and these could be used more frequently by therapists in future studies. Future trials may also need to increase the number of therapy sessions, as people with ARMS are often complex and may need more resourcing to prevent therapy feeling overwhelming, and then dropping out.

### Implications

There is a need to identify effective interventions that prevent transition to psychosis for people with ARMS. This study found that recruiting people with ARMS for a large multicentre RCT exclusively via the Early Intervention teams would be difficult in those Trusts which do not have a well-established pathway into care for people with ARMS, given the low number of people they identify. Therefore, future studies of people with ARMS may need to consider extending recruitment to primary care, Primary Care Liaison Services and the NHS Talking Therapies services in order to conduct trials in this area. Further work would be required to establish if recruiting via these routes is viable. In addition, involvement of other Trusts which have more established pathways into care for people with ARMS would be beneficial.

## Supporting information

Strelchuk et al. supplementary material Strelchuk et al. supplementary material

## Data Availability

The data that support the findings of this study are available from the corresponding author, D.S., upon reasonable request. The coding frames for the qualitative analyses are available from the corresponding author (D.S.) upon reasonable request. The authors assert that all procedures contributing to this work comply with the ethical standards of the relevant national and institutional committees on human experimentation and with the Helsinki Declaration of 1975, as revised in 2013. All procedures involving human subjects/patients were approved by the South-West Cornwall and Plymouth Research Ethics Committee (reference 18/SW/0037). Permissions to carry out the study were obtained from the NHS Trust involved.
