# Supplementary material for: Eye-movement desensitisation and reprocessing therapy (EMDR) to prevent transition to psychosis in people with an at-risk mental state (ARMS): mixed method feasibility study
Source: BJPsych Open. 2024 May 9;10(3):e105. doi: 10.1192/bjo.2024.57 (PMC11094432; doi:10.1192/bjo.2024.57)
Supplement: Strelchuk et al. supplementary material [file S2056472424000577sup001.docx]

**Supplement 1. Randomisation/blinding**

Randomisation took place using a remote (web-based) computerised randomisation service administered by the Bristol Trials Centre, and was minimized by psychotic symptom severity (score on positive symptoms on CAARMS <11 or ≥11). Researchers who carried out the follow-up assessments as part of the randomised study design were blinded to treatment allocation to avoid potential bias in outcome measurement.

**Supplement 2. Study eligibility**

To be eligible for the study, patients had to:

1. have experienced at least one traumatic event, before the onset of ARMS symptomatology, operationalized as:

a) a score of ≥ 3 on any item of The Life-Events Checklist for DSM-5 (LEC) (Gray *et al.*, 2004), or a score of ≥2 on items 12, 14, 15 or 16 of the LEC;

OR

b) a score of ≥ 9 on the Emotional Abuse Scale of the Childhood Trauma Questionnaire (CTQ) (Bernstein and Fink, 1998); or ≥ 8 on the Physical Abuse Scale; or ≥ 6 on the Sexual Abuse Scale; or ≥ 10 on the Emotional Neglect Scale; or ≥ 8 on the Physical Neglect Scale;

AND

1. have at least one PTSD symptom on the PTSD Checklist for DSM-5 (PCL-5) (Bovin *et al.*, 2016) operationalized as a score of ≥ 10 on the Re-experiencing subscale of the PCL-5; or ≥ 4 on the Avoidance subscale; or ≥ 14 on the Negative Thoughts subscale; or ≥ 12 on the Arousal and Reactivity subscale.

**Supplement 3. Study therapists**

Of the four EMDR therapists in our study, one therapist worked in an EI team, and the other three worked in other NHS services. Because of difficulties with securing excess treatment costs, therapists provided therapy as part of this study either (i) during their normal working hours; i.e. therapists in the study had to negotiate with their team managers to be allowed to see patients for the study within their normal working hours; or (ii) in a private capacity after we had received additional funding to pay for therapists’ time. Patients who were recruited from EI teams which were funded to work with ARMS had to complete the course of EMDR before they were discharged back to their GP. There were no time restrictions for those patients who were recruited from EI teams, which were not funded to work with ARMS.

The difficulties with securing excess treatment costs impacted therapists’ capacity to see patients, as part of the study, as most team managers only allowed therapists to see one patient at a time from their own EI team.

**Supplement 4. Clinical assessments**

CAARMS

The CAARMS has four subscales (unusual thought content, non-bizarre ideas, perceptual abnormalities and disorganised speech), and each subscale was scored on three dimensions: intensity, frequency and distress (on a scale from 0-6). Data presented in Table 2 was derived by averaging the scores of all subscales on each dimension. For example, CAARMS intensity was derived by averaging the intensity of unusual thought content, non-bizarre ideas, perceptual abnormalities and disorganised speech.

PSYRATS

Assessment of study participants started with the CAARMS and continued with the PSYRATS. As part of the PSYRATS, we only scored auditory hallucinations (i.e. not visual, tactile or other type) and delusions. If patients reported more than one auditory hallucination/delusion, the assessor rated the most severe experience patients had experienced in the week prior to assessment. If someone’s most severe experiences in the week prior to assessment were non-bizarre ideas of moderate intensity (e.g. mild paranoia), we rated those experiences of part of the PSYRAT-D.

CAPE

CAPE was adapted for use in this study, and participants were asked to rate their symptoms over the month prior to assessment.

**Supplement 5. Study challenges**

1. **Challenges to recruitment**

Our original feasibility study was designed as an RCT comparing EMDR with TAU, and the original recruitment target was 40 patients. However, recruitment to the study was difficult (e.g. during the first seven months of the study, we only recruited three participants against a target of 21). The poor recruitment was mainly due to the very low number of people identified as ARMS in the EI teams, and to some financial constraints in managing ARMS patients in EI teams, which meant that we were only able to recruit patients from three of the six EI teams in the Trust. We have therefore changed study design from randomised trial to single arm trial, and decreased the sample size to 20 patients. For more detail on challenges related to recruitment, please see our study protocol paper (Strelchuk et al, 2020).

1. **Impact of COVID-19 related restrictions**

The COVID-19 related restrictions impacted the study in the following ways:

1. Recruitment. Recruitment to the study, which was originally planned to close in April 2020, was further impacted by the COVID-19 pandemic as it coincided with an increase in the number of referrals with three referrals received just prior to the first national lockdown (March 2020). As it was unclear at the time when patients could be offered EMDR in-person, we extended recruitment for a month (until May 2020), hoping that we would get greater clarity on this by then. However, at the end of May there was no indication that these individuals could start EMDR in the foreseeable future, and therefore we decided not to offer those patients an eligibility baseline assessment.
2. Change in mode of delivering therapy. The change in the mode of delivering therapy from in-person to video-conferencing happened in October 2020, after NHS therapists had started to provide psychological therapies remotely. However, one participant did not agree to online EMDR which led to them dropping out of the study, and another patient opted to pause therapy until in-person EMDR was again available.
3. Change in the mode of conducting assessments. There was a change in the mode of conducting follow-up assessments (i.e. from in-person to over the telephone). The latter were more challenging due to technical issues, and/or participants finding it difficult to answer questions over the telephone. In addition, some questionnaires were posted to participants but not returned, which led to a substantial amount of missing data.

All these changes were discussed with our Trial Steering Committee (TSC) and reviewed by the Clinical Leadership and Oversight Group (CLOG) within AWP. These modifications were in place until the end of the study.

**Supplement 6. CONSERVE-CONSORT Extension**

| CONSERVE-CONSORT Extension: 3.05.2023 | | | | | |
| --- | --- | --- | --- | --- | --- |
| Item | Item Title | Description | | | Page No. |
| I. | Extenuating Circumstances | Describe the circumstances and how they constitute extenuating circumstances. | | | Supplement 5 |
| II. | Important Modifications | 1. Describe how the modifications are important modifications. | | | Supplement 5 |
|  |  | 1. Describe the impacts and mitigating strategies, including their rationale and implications for the trial. | | | Supplement 5 |
|  |  | 1. Provide a modification timeline. | | | Supplement 5 |
| III. | Responsible Parties | State who planned, reviewed and approved the modifications. | | | Supplement 5 |
| IV. | Interim data | If modifications were informed by trial data, describe how the interim data were used, including whether they were examined by study group, and whether the individuals reviewing the data were blinded to the treatment allocation. | | | N/A |
| CONSORT Number and Item | | For each row, if important modifications occurred check “direct impact” and/or “mitigating strategy” and describe the changes in the trial manuscript or supplement. Check “no change” for items that are unaffected in the extenuating circumstance. | | | Page No. |
|  |  | No Change | Impact* | Mitigating Strategy** |  |
| 1 | Title and abstract | x |  |  |  |
| 2 | Introduction | x |  |  |  |
| 3 | Methods: Trial Design | x |  |  |  |
| 4 | Methods: Participants | x |  |  |  |
| 5 | Methods: Interventions |  | x | x | Supplement 5 |
| 6 | Methods: Outcomes | x |  |  |  |
| 7 | Methods: Sample Size | x |  |  |  |
| 8-10 | Methods: Randomisation | x |  |  |  |
| 11 | Methods: Blinding | x |  |  |  |
| 12 | Methods: Statistical methods | x |  |  |  |
| 13 | Results: Participant flow | x |  |  |  |
| 14 | Results: Recruitment |  | x | x | Supplement 5 |
| 15 | Results: Baseline data | x |  |  |  |
| 16 | Results: Numbers analysed | x |  |  |  |
| 17 | Results: Outcomes and estimation | x |  |  |  |
| 18 | Results: Ancillary analyses | x |  |  |  |
| 19 | Results: Harms | x |  |  |  |
| 20 | Discussion: Limitations |  | x |  | Page 17-18 of the manuscript |
| 21 | Discussion: Generalisability | x |  |  |  |
| 23 | Other information: Registration | x |  |  |  |
| 24 | Other information: Protocol | x |  |  |  |
| 25 | Other information: Funding | x |  |  |  |
| *Aspects of the trial that are directly affected or changed by the extenuating circumstance and are not under the control of investigators, sponsor or funder.  **Aspects of the trial that are modified by the study investigators, sponsor or funder to respond to the extenuating circumstance or manage the direct impacts on the trial. | | | | | |

**Supplement 7. Psychotropic medication at baseline**

| Psychotropic medication | Randomised study | | | | | | Single arm study | | All participants who received EMDR | |
| --- | --- | --- | --- | --- | --- | --- | --- | --- | --- | --- |
|  | EMDR | | TAU | | Overall | |  |  |  |  |
|  | N (%) | Daily dose, mg (SD) | N (%) | Daily dose mg (SD) | N (%) | Daily dose mg (SD) | N (%) | Daily dose mg (SD) | N (%) | Daily dose mg (SD) |
| Any psychotropic medication: n (%) | 1 (33%) | - | 3 (100%) | - | 4 (67%) | - | 5 (71%)^1^ | - | 6 (60%)^2^ | - |
|  |  |  |  |  |  |  |  |  |  |  |
| Sertraline/Fluoxetine/Citalopram | 1 (100%) | 40 mg | 2 (67%) | 100 mg^3^ | 3 (75%) | 70 mg^4^ | 4 (80%) | 70 (34.6) | 5 (83%) | 64 (32.9) |
| Sertraline + Diazepam | - | - | 1 (33%) | 150mg + 10mg as needed | 1 (25%) | 150mg + 10mg as needed | - | - | - | - |
| Sertraline + Diazepam + Mirtazapine | - | - | - | - | - | - | 1 (20%) | 100mg + 5mg + 15 mg | 1 (17%) | 100mg + 5mg + 15 mg |

^1^7 of 8 participants had complete data on this measure; ^2^10 of 11 participants had complete data on this measure; ^3^1 of 2 participants had complete data on this measure; ^4^2 of 3 participants had complete data on this measure

**Supplement 8. Secondary outcome measures in participants who were offered EMDR (randomised and single arm trial)**

| Secondary outcomes | Timepoint | EMDR (combined) | | | TAU | | |
| --- | --- | --- | --- | --- | --- | --- | --- |
|  |  | Completed the measure | | Mean (SD) | Completed the measure | | Mean (SD) |
|  |  | N | % |  | N | % |  |
| CAARMS intensity | Baseline | 8 | 73% | 11.5 (1.1) | N/A^1^ |  |  |
|  | 4 months | 7 | 64% | 12.1 (1.3) | 1 | 33% | 12 |
|  | 8 months | 6 | 55% | 9.3 (1.1) | 2 | 67% | 4.5 (3.5) |
|  | 12 months | 6 | 55% | 8 (1.9) | 2 | 67% | 5 (3) |
| CAARMS frequency | Baseline | 8 | 73% | 11.6 (1.5) | N/A^1^ |  |  |
|  | 4 months | 7 | 64% | 9.9 (1.1) | 1 | 33% | 17 |
|  | 8 months | 6 | 55% | 9.5 (1.3) | 2 | 67% | 4.5 (3.5) |
|  | 12 months | 6 | 55% | 6.2 (1.3) | 2 | 67% | 5.5 (2.5) |
| CAARMS  distress | Baseline | 8 | 73% | 147.5 (28.7) | N/A^1^ |  |  |
|  | 4 months | 7 | 64% | 148.7 (26.1) | 1 | 33% | 90 |
|  | 8 months | 5 | 45% | 91 (17.1) | 2 | 67% | 34 (34) |
|  | 12 months | 5 | 45% | 138 (46.2) | 2 | 67% | 15 (15) |
| SOFAS | Baseline | 8 | 73% | 55.3 (4.6) | N/A^1^ |  |  |
|  | 4 months | 6 | 55% | 49.8 (3.3) | 1 | 33% | 85 |
|  | 8 months | 6 | 55% | 58.5 (4.9) | 2 | 67% | 85 (5) |
|  | 12 months | 6 | 55% | 64.5 (5.3) | 2 | 67% | 80.5 (0.5) |
| PSYRATS  Delusions | Baseline | 11 | 100% | 9.3 (1.6) | 3 | 100% | 7.3 (3.8) |
|  | 4 months | 7 | 64% | 12.1 (1.4) | 2 | 67% | 5.5 (5.5) |
|  | 8 months | 6 | 55% | 12.3 (1.3) | 2 | 67% | 3 (3) |
|  | 12 months | 6 | 55% | 7 (2.5) | 2 | 67% | 11.5 (2.5) |
| PSYRATS  Hallucinations | Baseline | 11 | 100% | 18.2 (4.4) | 3 | 100% | 20 (10.1) |
|  | 4 months | 7 | 64% | 14.1 (5.5) | 2 | 67% | 0 |
|  | 8 months | 6 | 55% | 12.3 (1.3) | 2 | 67% | 3 (3) |
|  | 12 months | 6 | 55% | 15.5 (5.1) | 2 | 67% | 0 |
| PANSS – negative scale | baseline | 11 | 100% | 15.5 (1.63) | 3 | 100% | 17 (1) |
|  | 4 months | 5 | 45% | 14.8 (2.42) | 2 | 67% | 11 (0) |
|  | 8 months | 6 | 55% | 12.2 (1.53) | 2 | 67% | 8 (1) |
|  | 12 months | 6 | 55% | 9 (0.8) | 2 | 67% | 9 (2) |
| CAPE-42 | Baseline | 10 | 91% | 39.1 (4.2) | 3 | 100% | 38 (4.6) |
|  | 4 months | 6 | 55% | 37.7 (3.9) | 2 | 66.7% | 38 (5) |
|  | 8 months | 6 | 55% | 32.5 (4.1) | 2 | 67% | 29.5 (9.5) |
|  | 12 months | 6 | 55% | 38.8 (5.1) | 2 | 67% | 28.5 (6.5) |
| CAPE-42 distress | Baseline | 10 | 91% | 26.4 (5.6) | 3 | 100% | 23 (3.5) |
|  | 4 months | 6 | 55% | 26.7 (6.6) | 2 | 67% | 18.5 (0.5) |
|  | 8 months | 6 | 55% | 18.7 (6.4) | 2 | 67% | 7.5 (7.5) |
|  | 12 months | 6 | 55% | 24.8 (8.9) | 2 | 67% | 8 (7) |
| PCL-5 | Baseline | 11 | 100% | 59.5 (3.2) | 3 | 100% | 52.7 (2.4) |
|  | 4 months | 6 | 55% | 41.2 (10.6) | 2 | 66.7% | 34.5 (6.5) |
|  | 8 months | 5 | 45% | 31.2 (5.9) | 2 | 67% | 17.5 (12.5) |
|  | 12 months | 7 | 64% | 41.9 (9.1) | 2 | 67% | 10.5 (10.5) |
| PHQ-9 | Baseline | 10 | 91% | 20 (1.7) | 3 | 100% | 16 (2) |
|  | 4 months | 6 | 55% | 14.3 (4.3) | 2 | 67% | 10 (6) |
|  | 8 months | 5 | 45% | 14.4 (3.6) | 2 | 67% | 6 (0) |
|  | 12 months | 5 | 45% | 14.6 (3.9) | 2 | 67% | 2.5 (1.5) |
| GAD-7 | Baseline | 10 | 91% | 14.6 (1.7) | 3 | 100% | 11.7 (3.5) |
|  | 4 months | 6 | 55% | 10.7 (3.4) | 2 | 67% | 6.5 (2.5) |
|  | 8 months | 5 | 45% | 10 (2.7) | 2 | 67% | 5 (2) |
|  | 12 months | 5 | 45% | 9.6 (3.4) | 2 | 67% | 2.5 (0.5) |
| WSAS | Baseline | 10 | 91% | 24.4 (1.1) | 3 | 100% | 16.3 (3.5) |
|  | 4 months | 6 | 55% | 22 (5.0) | 2 | 66.7% | 11.5 (0.5) |
|  | 8 months | 5 | 45% | 22.6 (5.7) | 2 | 67% | 6.5 (3.5) |
|  | 12 months | 5 | 45% | 19.2 (5.6) | 2 | 67% | 5 (3) |
| EQ-5D-L | Baseline | 10 | 91% | 11 (0.7) | 3 | 100% | 10.3 (1.5) |
|  | 4 months | 6 | 55% | 11.7 (2.4) | 2 | 66.7% | 6.5 (1.5) |
|  | 8 months | 5 | 45% | 10.2 (1.8) | 2 | 67% | 6 (0) |
|  | 12 months | 5 | 45% | 11.8 (2.6) | 2 | 67% | 6 (1) |
| DAST-10 (Yes/No) |  | n | % | n (%Yes) | n | % | n (% Yes) |
|  | Baseline | 10 | 91% | 3 (30%) | 3 | 100% | 2 (67%) |
|  | 4 months | 5 | 45% | 2 (40%) | 2 | 67% | 2 (100%) |
|  | 8 months | 4 | 36% | 1 (25%) | 2 | 67% | 2 (100%) |
|  | 12 months | 5 | 45% | 2 (40%) | 2 | 67% | 1 (50%) |

^1^These measures were not collected at baseline

**Supplement 9. Psychotropic medication at follow-up in participants who were offered EMDR and participants who received TAU**

|  | EMDR (combined) | | TAU | |
| --- | --- | --- | --- | --- |
| Psychotropic medication | N(%) | Daily dose (mg)  (mean (SD)) | N(%) | Daily dose (mg)  (mean (SD)) |
| 4-month FU  Any psychotropic medication | 4 (67%)^1^ | - | 0^2^ | - |
| Sertraline/Fluoxetine/ Citalopram | 3 (50%) | 30 (17.3) | 0 | - |
| Sertraline + Diazepam + Mirtazapine | 1 (17%) | 100mg + 5 mg (as needed) + 15 mg | 0 | - |
| 8-month FU  Any psychotropic medication | 4 (80%)^3^ | - | 1 (50%)^4^ | - |
| Sertraline/Fluoxetine/ Citalopram | 3 (60%) | 33.3 (11.5) | 1 (50%) | 20 mg |
| Sertraline + Diazepam + Mirtazapine | 1 (20%) | 100 mg + 5 mg (as needed) + 15 mg | 0 | - |
| 12-month FU  Any psychotropic medication | 4 (57%)^5^ | - | 1 (50%)^4^ | - |
| Sertraline/Fluoxetine/Citalopram | 2 (33%) | 50 (14.1) | 1 (50%) | 20 mg |
| Mirtazapine | 1 (17%) | 70 mg | - | - |
| Sertraline + Diazepam + Mirtazapine | 1 (17%) | 100 mg + 5 mg (as needed) + 15 mg | - | - |

^1^6 of 11 participants had complete data; ^2^2 of 3 participants had complete data; ^3^5 of 11 had complete data; ^4^2 of 3 had complete data; ^5^7 of 11 participants had complete data

**Supplement 10. Use of services in participants who were offered EMDR and TAU**

| Resource use | EMDR  (randomised and single arm study) | | | TAU | | |
| --- | --- | --- | --- | --- | --- | --- |
|  | 4-month FU | 8-month FU | 12-month FU | 4-month FU | 8-month FU | 12-month FU |
| **Treatment for mental health**  (N who had the treatment, number of sessions) |  |  |  |  |  |  |
| **N completed question (%)** | 5 (45%) | 3 (27%) | 6 (55%) | 2 (67%) | 1 (33%) | 2 (67%) |
| EMDR therapy (separate from what was received as part of the study) |  |  |  |  |  |  |
| N who had the treatment  Mean number of sessions (SD) | 0  - | 0  - | 0  - | 0  - | 0  - | 0  - |
| CBT  N who had the treatment  mean number of sessions (SD) | 0  N/A | 1  missing | 1  12 | 1  4 | 1  10 | 0  - |
| Family intervention  N who had the treatment  Mean number of sessions (SD) | 1  3 | 0  - | 0  - | 0  - | 0  - | 0  - |
| Counselling:  N who had the treatment  mean number of sessions (SD) | 1  6 | 0  - | 0  - | 2  6 (4) | 1  3 | 0  - |
| Education about mental health:  N who had the treatment  Mean number of sessions (SD) | 0  - | 1  missing | 0  - | 1  30 | 1  9 | 1  missing |
| **GP appointments** |  |  |  |  |  |  |
| **N completed question (%)** | 5 (45%) | 5 (45%) | 6 (55%) | 2 (67%) | 2 (67%) | 2 (67%) |
| Number of GP visits: mean (SD) | 1 (0.45) | 1 (0.45) | 0.33 (0.2) | 1.5 (1.5) | 2.5 (2.5) | 2 (2) |
| “Out of hours” GP visits: mean (SD); (N completed if different from above, %) | 0; (4, 36%) | 0.2 (0.2) | 0.2 (0.2) | 0; (1, 33%) | 0 | 0.5 (0.5) |
| NHS secondary care - for mental health (N who accessed this service, number of visits) |  |  |  |  |  |  |
| **N completed question (%)** | 5 (45%) | 4 (36%) | 6 (55%) | 2 (67%) | 2 (67%) | 2 (67%) |
| Early Intervention Team:  N who accessed this service  Mean number of visits (SD); (N completed if different from N who accessed this service, %) | 2  8; (1, 50%) | 3  1; (1, 33%) | 2  3 (1) | 1  10 | 1  10 | 0  - |
| NHS outpatient or community mental health clinics:  N who accessed this service  mean number of visits (SD); (N completed if different from above, %) | 0  - | 2  2 (1, 50%) | 0  - | 0  - | 0  - | 0  - |
| A&E department:  N who accessed this service  mean number of visits (SD) | 0  - | 0  - | 0  - | 0  - | 0  - | 1  Missing data |
| Other services - for any medical condition (number of visits) |  |  |  |  |  |  |
| **N completed question (%)** | 5 (45%) | 5 (45%) | 6 (55%) | 2 (67%) | 2 (67%) | 2 (67%) |
| Complementary therapy:  N who had the treatment  mean number of visits (SD) | 0  - | 0  - | 0  - | 1  2 | 1  3 | 1  2 |
| Physical activity scheme:  N who had the treatment  mean number of visits (SD) | 0  - | 0  - | 0  - | 0  - | 0  - | 0  - |
| NHS Walk-in Centre:  N who had the treatment  Mean number of visits (SD) | 0  - | 0  - | 1  1 | 0  - | 0  - | 0  - |
| Additional help - because of mental health difficulties |  |  |  |  |  |  |
| **N completed question (%)** | 5 (45%) | 5 (45%) | 6 (55%) | 2 (67%) | 2 (67%) | 2 (67%) |
| Help with school:  N who needed help | 1 | 1 | 1 | 0 | 0 | 0 |
| Help in the house:  N who needed help | 2 | 3 | 4 | 0 | 0 | 0 |
| Help outside the house:  N who needed help | 2 | 2 | 2 | 0 | 0 | 0 |
| Time off school or work - because of mental health (N who needed time-off, number of days) |  |  |  |  |  |  |
| **N completed question (%)** | 5 (45%) | 5 (45%) | 6 (55%) | 2 (67%) | 2 (67%) | 2 (67%) |
| Patient:  N who needed time off  mean number of days (SD); (N completed if different from above, %) | 3  2.5 (1.5); (2, 67%) | 3  20; (1, 33%) | 2  16; (1, 50%) | 2  7; (1, 50%) | 1  4 | 0  - |
| Carer:  N who had to take time off  mean number of days (SD) | 0  - | 0  - | 0  - | 0  - | 0  - | 0  - |
| Home visits - for any condition (N who accessed service, number of visits) |  |  |  |  |  |  |
| **N completed question (%)** | 5 (45%) | 5 (45%) | 6 (55%) | 2 (67%) | 2 (67%) | 2 (67%) |
| Mental health nurse:  N who accessed service  mean number of visits (SD) | 0  - | 1  Missing data | 0  - | 0  - | 0  - | 0  - |
| Occupational therapist:  N who accessed service  mean number of visits (SD) | 0  - | 0  - | 0  - | 0  - | 0  - | 0  - |
| Social worker:  N who accessed service  mean number of visits (SD) | 0  - | 0  - | 0  - | 0  - | 0  - | 0  - |
| GP:  N who accessed service  mean number of visits (SD) | 0  - | 0  - | 0  - | 0  - | 0  - | 0  - |
| Additional help - for mental health (yes/no) |  |  |  |  |  |  |
| **N completed question (%)** | 5 (45%) | 4 (36%) | 6 (55%) | 2 (67%) | 2 (67%) | 2 (67%) |
| Home care worker:  N who accessed service | 0 | 0 | 0 | 0 | 0 | 0 |
| Day centre:  N who accessed service | 0 | 1 | 0 | 0 | 0 | 0 |
| Self-help group:  N who accessed service | 0 | 1 | 0 | 0 | 1 | 1 |
| Voluntary organisation:  N who accessed service | 1 | 0 | 0 | 0 | 0 | 0 |
| Disability payments |  |  |  |  |  |  |
| **N completed question (%)** | 5 (45%) | 5 (45%) | 5 (45%) | 2 (67%) | 2 (67%) | 2 (67%) |
| NHS prescription charges:  N who accessed service | 2 | 2 | 2 | 2 | 2 | 2 |
| Disability payments:  N who accessed service | 3 | 3 | 3 | 0 | 0 | 0 |

### Supplement 11. Adverse events

|  | TAU | | | | | | All participants who were offered EMDR | | | | | |
| --- | --- | --- | --- | --- | --- | --- | --- | --- | --- | --- | --- | --- |
|  | Events | | Patients | | | | Events | | Patients | | | |
|  | AE | SAE | AE | % | SAE | % | AE | SAE | AE | % | SAE | % |
| Overdose/self-harm | 0 | 0 | 0 | - | 0 | - | 1 | 1 | 1 | 9 | 1 | 9 |
| Risk of harm to others | 0 | 0 | 0 | - | 0 | - | 1 | 0 | 1 | 9 | 0 | - |

**Supplement 12. Challenges faced in our study and recommendations for future ARMS trials**

|  | Challenges faced in our study | Recommendations for future ARMS trials |
| --- | --- | --- |
| Eligibility criteria and difficulties meeting recruitment target | Our exclusion criteria of not offering patients EMDR concurrently with other psychological therapies (e.g. CBTp) negatively impacted recruitment at the start of the study. This was because, due to difficulties with securing excess treatment costs, at the start of the study, we were only able to recruit from those EI teams which were funded to work with ARMS. As those EI teams offered patients CBTp as part of TAU, most patients would have started CBTp by the time we opened recruitment, and were therefore ineligible for the trial.  However, as the study progressed and those EI teams took on new ARMS patients, the EI teams agreed to hold the delivery of CBTp until after our research team had conducted the eligibility assessment, so that eligible patients could be offered EMDR. Recruitment was further improved from six months in once we obtained funding to pay therapists working in a private capacity, enabling us to recruit from those EI teams that were not funded to work with ARMS. However, given the small number of ARMS patients in the participating EI teams, we were unable to meet our recruitment target. | As the number of ARMS patients in the EI teams in the UK is generally small, we recommend that future ARMS trials extend recruitment to other services (e.g. PCLS and IAPT).  We think that future ARMS trials should continue to investigate the effectiveness of EMDR separately from other psychological interventions, as by offering clients EMDR and CBTp in parallel it would be difficult to disentangle the impact of the two interventions. In addition, evaluating EMDR as an adjunct to CBTp would require a substantially larger sample size compared to evaluating EMDR alone.  Furthermore, we think that offering patients concomitantly two psychological interventions such as EMDR and CBTp would not usually happen in clinical practice, and therefore, the findings of such a trial would be difficult to generalise to current clinical practice.  The impact of this exclusion criterion on recruiting participants from EI teams where CBTp is offered as part of TAU can be minimised by ensuring a close liaison between researchers and clinical teams, so that patients have the option to participate in a trial in a timely fashion. Clinical teams could still offer other psychological therapies (e.g. CBTp) after completion of EMDR, if required.  Furthermore, given that EI clinicians are very busy, if would be helpful for future trials to hire a clinician from the clinical team, who would have direct responsibility for recruiting participants.  If this was not possible, then it would be important to ensure that researchers are based within clinical teams in order to raise the profile of the study to clinicians |
| Clinical assessments | There was a substantial amount of missing data in the follow-up assessments. Although the original plan was that all follow-up assessments would be conducted in-person, in the context of the COVID-19 restrictions, these were conducted over the telephone. The latter were more challenging due to technical issues (around connections/signal), and/or participants finding it difficult to answer questions over the telephone, which may have contributed to the substantial amount of missing data. | Including the option of doing videocall assessments may increase participant engagement, although researchers need to ensure that participants would have a device to enable this. Giving participants the option of filling out questionnaires online (by using tools such as the Qualtrics) may also improve completion rates in future studies. |
| EMDR protocol | Some therapists said that EMDR would have been more effective had patients been offered more therapy sessions. Some therapists also said that their patients did not have the skills to regulate the distressing emotions associated with trauma processing. | A pragmatic approach for future trials would be to allow more flexibility around the number of EMDR sessions (e.g. up to 20 sessions), so that therapists could spend more time on enhancing patients’ emotion regulation skills, and offering those with attachment difficulties additional preparatory work. This would also give therapists more confidence that they have sufficient time to address multiple/highly-complex traumas if required.  Although therapists were trained in using a number of emotion-regulation techniques, in future trials having a broader range of techniques to regulate emotions and reduce the distress around trauma processing would be helpful. The use of techniques such as the EMDR bomb or the flash technique could facilitate the use of EMDR even when patients initially found their memories too overwhelming to allow processing to occur. |
| Fidelity assessment of EMDR sessions | The independent assessor indicated that the scale used for assessing fidelity was quite laborious, and some of the items could not be scored even though it was likely that they were covered in a previous session. | Future trials may need to use a simplified version of the scale, which will have to be adapted for the purpose of fidelity assessment as part of a clinical trial. |
